# Prospective evaluation of the prevalence and characteristics of heart murmurs and gallop sounds in anemic cats

**DOI:** 10.1093/jvimsj/aalag261

**Published:** 2026-10-24

**Authors:** Alison Robertson, Adrian Boswood, Virginia Luis Fuentes, Eve J Y Lo, Karen Humm

**Affiliations:** Department of Clinical Science and Services, Royal Veterinary College, Hatfield AL9 7TA, United Kingdom; Department of Clinical Science and Services, Royal Veterinary College, Hatfield AL9 7TA, United Kingdom; Department of Clinical Science and Services, Royal Veterinary College, Hatfield AL9 7TA, United Kingdom; Department of Clinical Science and Services, Royal Veterinary College, Hatfield AL9 7TA, United Kingdom; Department of Clinical Science and Services, Royal Veterinary College, Hatfield AL9 7TA, United Kingdom

**Keywords:** anemia, auscultation, blood transfusion, cardiac disease, cardiomyopathy, feline

## Abstract

**Background:**

Heart murmurs and gallop sounds are common in anemic cats; however, their association with underlying cardiac disease is unclear.

**Hypothesis/Objectives:**

Determine the prevalence of murmurs and gallops in anemic cats. Investigate the association of auscultatory abnormalities with anemia severity and heart disease. Investigate whether change in anemia severity affects the audibility of murmurs and gallop sounds.

**Animals:**

Sixty-four anemic cats (packed cell volume [PCV] below 30%) presenting to a referral hospital.

**Methods:**

Single-center prospective observational study. On presentation, cats were examined and had PCV measurement. Each cat underwent echocardiography. Cats had PCV checks and daily auscultation to document heart sounds.

**Results:**

Fifty-eight percent (37/64) had a murmur and 23% (15/64) had a gallop. Mean PCV at enrollment was 18% (±6). Lower PCV was associated with the presence of a murmur (*P* = .03) and gallop (*P* = .016). Cardiac disease was present in 34% (22/64) of cats. After adjusting for anemia severity, murmur presence showed no statistically significant association with cardiac disease (*P* = .636 (OR: 1.31, 95% CI, 0.43-3.95)). A gallop sound was associated with greater odds of cardiac disease (*P* = .037 (OR: 3.97, 95% CI, 1.09-14.5)). Murmur intensity (*P* = .001) and likelihood of a gallop sound (*P* = .022) were lower in individuals when their PCVs increased.

**Conclusions and clinical importance:**

Murmurs and gallop sounds are common in anemic cats with and without cardiac disease. Anemic cats with gallops are more likely to have underlying cardiac disease than anemic cats without gallops.

## Introduction

Heart murmurs and gallop sounds are frequently present in anemic cats, with 30%-60% reported to have a heart murmur.^1–4^ Previous studies have given widely differing estimates of the prevalence of a gallop sound in anemic cats (5.3%-60%).^1–4^ A retrospective study assessing heart sounds in cats presenting as emergencies found that heart murmurs and gallop sounds are more common in anemic cats than non-anemic cats.^3^ However, in that study cohort, underlying cardiac disease was not excluded as a cause for the auscultatory abnormalities detected.

Heart murmurs are present in 30%-51% of apparently healthy cats.^5–9^ Cats might have a heart murmur due to structural heart disease, such as hypertrophic cardiomyopathy (HCM), which is reported in 11.0%-19.6% of clinically healthy cats, however, heart murmurs are frequently found in cats without evidence of underlying heart disease.^8^^,^^10–12^ There is a wide reported range of prevalence of gallop sounds auscultated in cats with cardiomyopathies (2.6%-40%).^7^^,^^13^

A previous study assessing echocardiographic changes in anemic cats revealed 70% of cats had signs of volume overload before transfusion.^13^ Repeat echocardiography after transfusion found fractional shortening decreased and left ventricular internal diameter in systole increased significantly. Prevalence of changes consistent with volume overload did not significantly change after transfusion; 60% of cats had evidence of volume overload after transfusion.^14^ No previous studies have described the temporal change in heart murmurs or gallop sounds in anemic cats. Anecdotally, murmur intensity appears to reduce in individual anemic cats with an increase in their packed cell volume (PCV).

This prospective study aimed to characterize abnormal heart sounds in anemic cats. We hypothesized that heart murmurs and gallop sounds would be common in anemic cats and that auscultatory abnormalities would occur more frequently in cats with worse anemia. We also hypothesized that heart murmur intensity would decrease if an individual cat’s PCV increased over time and that gallop sounds would resolve. The study also aimed to determine the prevalence of echocardiographic abnormalities in a cohort of anemic cats, whether any characteristics differed between anemic cats with cardiac disease and those with a normal heart, and if any characteristics of the cats were associated with survival to discharge.

## Materials and methods

Cats were prospectively recruited in a referral hospital over 1 year. Anemic cats (PCV < 30%) without pre-existing cardiac disease or hyperthyroidism were eligible for enrollment. Cats were excluded if owners withdrew consent, echocardiography could not be interpreted in light of systemic disease, they were intolerant of auscultation and/or echocardiography, or if they were discharged/died before echocardiography (Figure 1).

**Figure 1:**
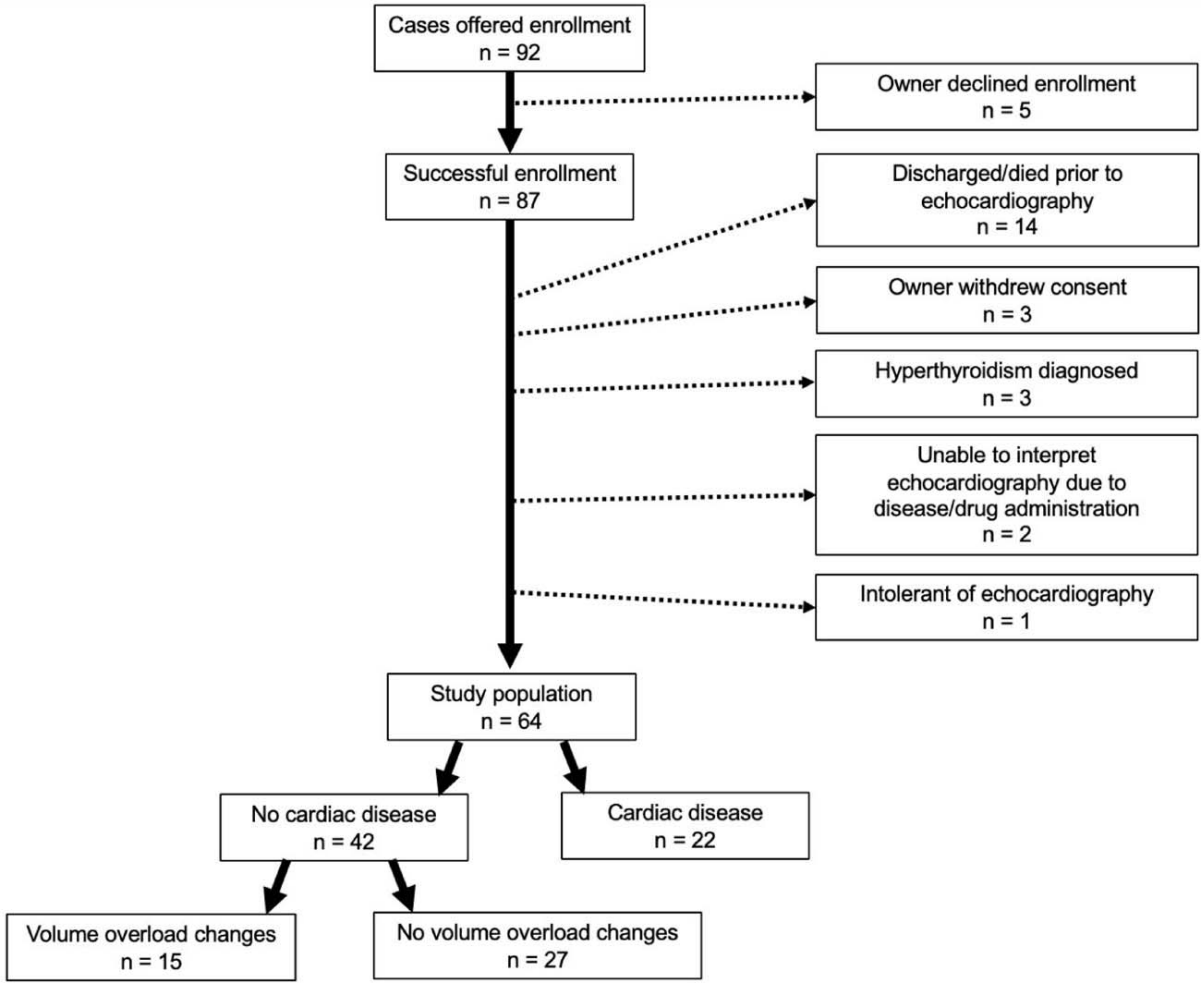
Case enrollment.

Cardiac auscultation was performed by the primary investigator once daily. Each cat had echocardiography performed once by a board-certified cardiologist or resident-in-training. HCM phenotype, equivocal HCM phenotype, and left ventricular dilation were defined and staged as described.^7^^,^^15^ Cats with evidence of a cardiomyopathy were placed in the “cardiac disease” group. Volume overload (high cardiac output) changes were considered as secondary to anemia; these cases were included in the “no cardiac disease” group.^13^

Auscultation was performed each day and the following were recorded: PCV, murmur grade (0-VI), and presence/absence of a gallop. For each cat with ≥2 PCV values recorded over time, the difference between the highest and lowest PCV was termed “δ PCV.” Murmur grade and presence/absence of a gallop at the highest and lowest PCV was compared. The cause of anemia was classified as either blood loss, hemolysis, ineffective production, or chronic disease. Outcome was survival to discharge or death during hospitalization.

Data distribution was assessed using the Shapiro–Wilk test. Continuous variables were reported as mean (±SD) if normally distributed or median (range) if not. Continuous and ordinal data were compared using independent samples *t-*test or Mann–Whitney U test. Associations between categorical variables were assessed using χ-squared or Fisher’s exact test.

Binary logistic regression analyses were undertaken, one with the presence of a gallop as the outcome variable and the other with the presence of a murmur as the outcome variable. In both, admission PCV and the presence/absence of heart disease were used as predictors.

Receiver operator curve (ROC) analysis was performed to assess for cut-off admission PCV predictive of a murmur or gallop. Area under the curve was calculated and optimal cut-off was determined by Youden’s index. If auscultation and PCV were recorded at least twice, observations at the lowest PCV were compared to those at the highest PCV. Murmur grade was compared using Wilcoxon Signed Rank test and the presence of a gallop sound was compared using McNemar test. Significance was set as *P*-value <.05. Further information is provided in Appendix.

## Results

### Case demographics

A total of 92 owners were offered enrollment of their cat during the study period. After owners declined enrollment, withdrew consent, or cases were excluded, 64 cats were included in the study (Figure 1). The breeds represented included Domestic Shorthair (59%, 38/64), Domestic Longhair (13%, 8/64), Maine Coon, Ragdoll, and British Shorthair (6%, 4/64 each), European Shorthair (3%, 2/64), and one each of: Norwegian Forest cat, Burmese, Sphynx, and crossbreed. The median age of cats at enrollment was 64 months (range 7-211). The mean body weight of cats was 4 kg (±1) and median body condition score was 4 out of 9 (range 1-8). At enrollment the mean PCV was 18% (±6) and mean heart rate was 190 beats per minute (±26). The median lowest PCV reported at any time point was 15% (range 6-29). Causes of anemia were: anemia of chronic disease (36%, 23/64), ineffective production (30%, 19/64), blood loss (23%, 15/64), and hemolysis (11%, 7/64).

### Auscultatory abnormalities

At the time of enrollment in the study 58% (37/64; 95% CI, 45-70) cats had a heart murmur, the median murmur grade was II/VI (range 0-IV; Figure 3). At the time of each cat’s lowest PCV, 67% (43/64; 95% CI, 54-78) had a murmur and the median murmur grade was II/VI (range 0-IV). Lower PCV at enrollment (*P* = .026; Figure 2), increased left atrial:aorta (left atrial to aorta ratio [LA:Ao]; *P* = .036) and having a gallop sound (*P* = .015) were significantly associated with the presence of a heart murmur (Table 1). The results of binary logistic regression (Table 2) showed that for each 1% increase in PCV the odds of having a heart murmur decreased (OR: 0.911, 95% CI, 0.836-0.993 (*P* = .034)). However, no statistically significant association was detected between the presence of cardiac disease and the presence of a murmur (OR: 1.306, 95% CI, 0.43-3.95, *P* = .636); given the wide confidence interval, this finding does not rule out a clinically relevant association (see Discussion section). The ROC analysis of the relationship between PCV and the presence/absence of a heart murmur revealed an area under the curve (AUC) of 0.66 (95% CI, 0.53-0.80) with no optimal cut-off value. Undertaking ROC analysis using the predicted probabilities from the logistic regression model did not improve the AUC (0.67 (95% CI, 0.54-0.81)).

**Figure 2:**
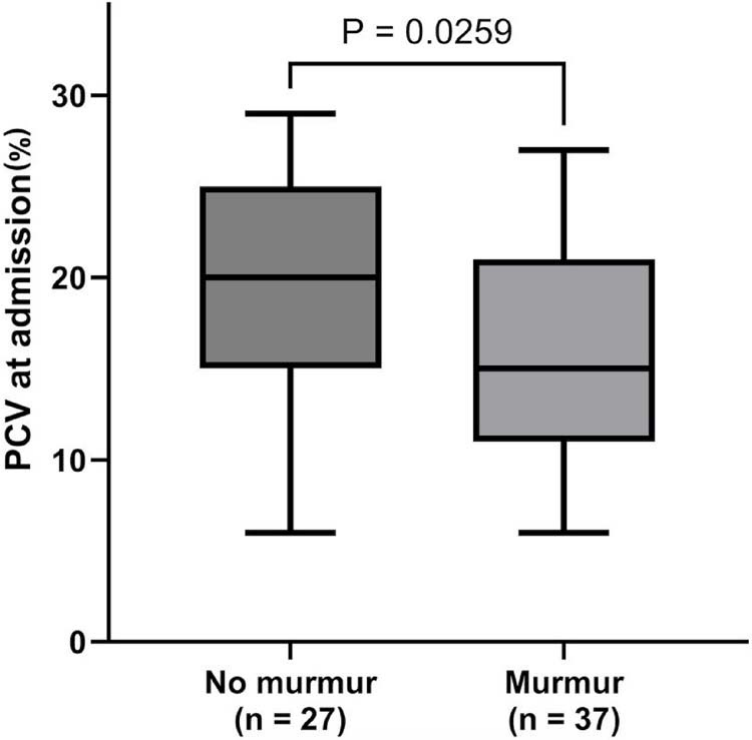
Box and whisker plots comparing the PCV in cats with and without a heart murmur. Box and whisker plots display the median (horizontal line within the box), interquartile range (box, 25th-75th percentile) and overall range (whiskers) for PCV. Median PCV was significantly lower in cat with murmurs than without murmurs. Abbreviation: PCV = packed cell volume.

**Figure 3:**
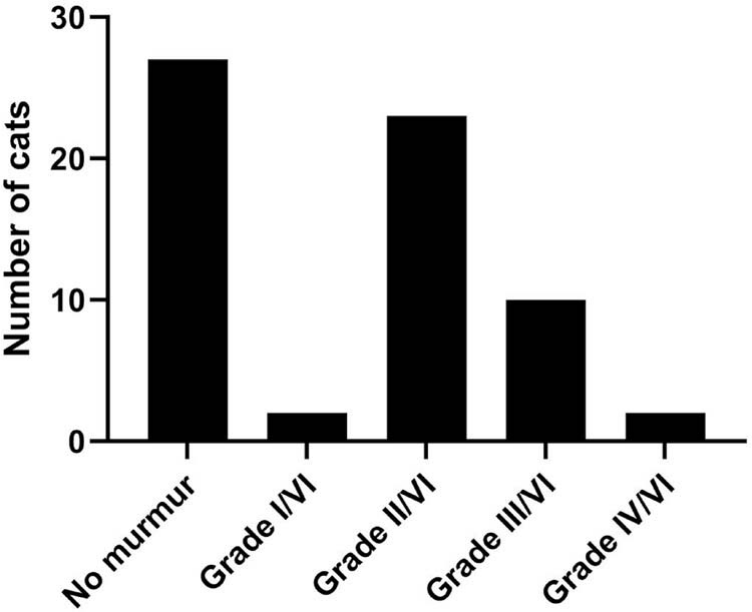
Murmur grade at admission to the study.

**Table 1:** Variables and their association with the presence of a heart murmur.

Variable	Murmur (*n* = 37)	No murmur (*n* = 27)	*P*-value
**Age (months) [Median (range)]**	77 (12-211)	36 (7-192)	.123
**Bodyweight (kg) [Mean ± SD]**	4.26 ± 0.93	4.01 ± 1.25	.374
**PCV (%) [Median (range)]**	15 (6-27)	20 (6-29)	***.026***
**LA:Ao [Mean ± SD]**	1.55 ± 0.28 (*n* = 30)	1.40 ± 0.16 (*n* = 24)	***.036***
**Heart rate (bpm) [Mean ± SD]**	193 ± 24	187 ± 29	.337
**Cardiac disease**	38% (14/37) (95% CI, 23-55)	30% (8/27) (95% CI, 14-50)	.598
**Gallop**	35% (13/37) (95% CI, 20-53)	7.4% (2/27) (95% CI, 0.9-24.3)	***.015***
**Survived to discharge**	78% (29/37) (95% CI, 62-90)	70% (19/27) (95% CI, 50-86)	.563

Data are presented as mean±SD for normally distributed variables and median (range) for non-normally distributed variables. Abbreviations: SD = standard deviation; LA:Ao = left atrial to aorta ratio; bpm = beats per minute; 95% CI = 95% confidence interval.

**Table 2:** Results of the binary logistic regression analysis with the presence of a murmur (yes/no) as the outcome and PCV at admission and the presence of cardiac disease as the predictor variables.

						95% CI	
Predictor	Estimate	SE	Z-statistic	*P*-value	Odds ratio (OR)	Lower	Upper
**Intercept**	1.8947	0.8712	2.175	.030	6.650	1.206	36.677
**PCV at admission**	−0.0930	0.0438	−2.125	***.034***	0.911	0.836	0.993
**Cardiac disease: Yes/No**	0.2672	0.5646	0.473	.636	1.306	0.432	3.951

Abbreviations: PCV = packed cell volume; SE = standard error; 95% CI = 95% confidence interval.

A gallop sound was present in 23% (15/64; 95% CI, 14-36) cats at enrollment in the study. At the time of each cat’s lowest PCV, 34% (22/64; 95% CI, 23-47) had a gallop sound. Lower PCV at enrollment (*P* = .006; Figure 4), increased LA:Ao (*P* = .014), heart rate (*P* ≤ .001), presence of cardiac disease (*P* = .028), and presence of a heart murmur (*P* = .015) were significantly associated with the presence of a gallop sound (Table 3). The results of binary logistic regression (Table 4) showed that for each 1% increase in PCV the odds of having a gallop sound decreased (OR: 0.871, 95% CI, 0.78-0.98 (*P* = .016)) and the presence of cardiac disease was associated with higher odds of having a gallop sound (OR 3.97, 95% CI, 1.09-14.5 (*P* = .037)). ROC analysis of the relationship between PCV and the presence/absence of a gallop revealed an AUC of 0.74 (95% CI, 0.61-0.88), suggesting that PCV had moderate discriminative ability to predict the presence of a gallop sound. The optimal cut-off value was a PCV of 20%, which had a sensitivity of 87% (95% CI, 62-96) and specificity of 43% (95% CI, 30-57) for prediction of the presence of a gallop. Undertaking ROC analysis using the predicted probabilities from the logistic regression model resulted in a higher AUC of 0.82 (95% CI, 0.72-0.92).

**Figure 4:**
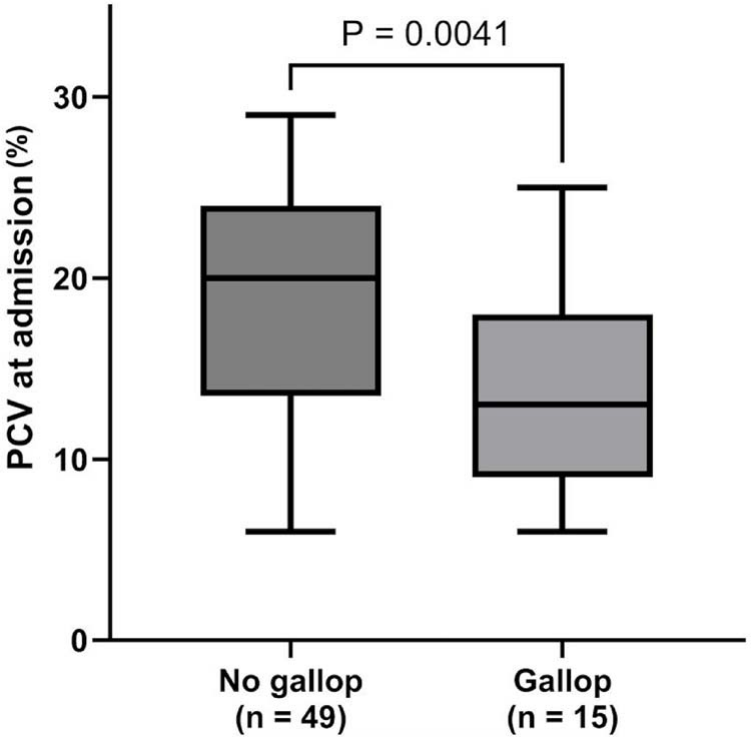
Box and whisker plots comparing the PCV in cats with and without a gallop sound. Box and whisker plots display the median (horizontal line within the box), interquartile range (box, 25th-75th percentile) and overall range (whiskers) for PCV. Median PCV was significantly lower in cat with gallops than without gallops. Abbreviation: PCV = packed cell volume.

**Table 3:** Variables and their association with the presence of a gallop sound.

Variable	Gallop (*n* = 15)	No gallop (*n* = 49)	*P*-value
**Age (months) [Median (range)]**	33 (12-168)	73 (7-211)	.307
**Bodyweight (kg) [Median (range)]**	4.55 (2.69-6.10)	3.90 (1.75-6.9)	.094
**PCV (%) [Mean ± SD]**	14 ± 5	19 ± 6	***.006***
**LA:Ao [Median (range)]**	1.60 (1.34-2.02) (*n* = 12)	1.40 (1.06-2.31) (*n* = 42)	***.014***
**Heart rate (bpm) [Mean ± SD]**	210 ± 22.8	184 ± 24.1	***≤.001***
**Cardiac disease**	60.0% (9/15) (95% CI, 32-84)	27% (13/49) (95% CI, 15-41)	***.028***
**Heart murmur**	87% (13/15) (95% CI, 60-98)	49% (24/49) (95% CI, 34-64)	***.015***
**Survived to discharge**	80% (12/15) (95% CI, 52-96)	74% (36/49) (95% CI, 59-85)	.742

Data are presented as mean±SD for normally distributed variables and median (range) for nonnormally distributed variables. Abbreviations: SD = standard deviation; LA:Ao = left atrial to aorta ratio; bpm = beats per minute; 95% CI = 95% confidence interval.

**Table 4:** Results of the binary logistic regression analysis with the presence of a gallop sound (yes/no) as the outcome and PCV at admission and the presence of cardiac disease as the predictor variables.

						95% CI	
Predictor	Estimate	SE	Z-statistic	*P*-value	Odds ratio (OR)	Lower	Upper
**Intercept**	0.486	0.9727	0.500	.617	1.627	0.242	10.945
**PCV at admission**	−0.139	0.0578	−2.401	***.016***	0.871	0.777	0.975
**Cardiac disease: Yes/No**	1.379	0.6608	2.087	***.037***	3.972	1.088	14.504

Abbreviations: PCV = packed cell volume; SE = standard error; 95% CI = 95% confidence interval.

### Echocardiography findings

34% (22/64; 95% CI, 23-47) of cats were considered to have cardiac disease. A HCM phenotype was present in 55% (12/22; 95% CI, 32-76) of these cats, 41% (9/22; 95% CI, 21-64) had equivocal HCM and 5% (1/22; 95% CI, 0-23) had restrictive cardiomyopathy. Volume overload changes were present in 36% (15/42; 95% CI, 22-52) of cats placed in the no cardiac disease group.

In cats with cardiac disease, 64% (14/22; 95% CI, 41-83) had a murmur and 41% (9/22; 95% CI, 21-64) had a gallop. Of the cats in the no cardiac disease group, 55% (23/42; 95% CI, 39-70) had a heart murmur and 14% (6/42; 95% CI, 5-29) had a gallop sound. In cats in the no cardiac disease group with volume overload changes, 73% (11/15; 95% CI, 45-92) had a heart murmur and 20% (3/15; 95% CI, 4-48) had a gallop sound. In cats with neither volume overload nor cardiac disease 44% (12/27; 95% CI, 26-65) had a heart murmur and 11% (3/27; 95% CI, 2-29) had a gallop sound (Table 5).

**Table 5:** Number and proportion, with 95% CI of cats with different echocardiographic classifications found to have a murmur or gallop sound.

	Murmur	Gallop
**Cardiac disease (*n* = 22)**	64% (14/22) (95% CI, 41-83)	41% (9/22) (95% CI, 21-64)
**Volume overload (*n* = 15)**	73% (11/15) (95% CI, 45-92)	20% (3/15) (95% CI, 4-48)
**Normal without evidence of volume overload (*n* = 27)**	44% (12/27) (95% CI, 26-65)	11.1% (3/27) (95% CI, 2-29)
**Total**	58% (37/64) (95% CI, 46-69)	23% (15/64) (95% CI, 14-36)

In cats in the no cardiac disease group, the presence of a heart murmur (*P* = .11) or gallop sound (*P* = .65) were not associated with having volume overload changes. Trivial valvular insufficiency was present in 20% (3/15; 95% CI, 4-48) cats with volume overload changes. These cats had both pulmonic and tricuspid regurgitation (*n* = 2) or tricuspid regurgitation alone (*n* = 1).

### Change in auscultatory abnormalities over time

Serial data were recorded in 70% (45/64) of cats. The median of the maximal change in PCV over time was 6% (range 0-18). Thirty-two of these cats had a murmur audible at the time of the lowest PCV and 25 had a murmur audible at the time of the highest PCV. Audible murmur grade tended to be lower when cats had a higher PCV (*P* = .001). In 38% (17/45) cats (95% CI, 24-54) cats the murmur grade was lower at the time of the highest PCV, in 56% (25/45) cats (95% CI, 40-70) the murmur grade remained the same (including 12 cats that had no murmur audible on both occasions) and in 7% (3/45) cats (95% CI, 1-18) the murmur grade was higher at the time of the highest PCV. Cats were more likely to have a gallop audible at the time of their lowest PCV (OR: 5.5 [95% CI, 1.2-51.1] (*P* = .022)). The presence or absence of gallop sounds at the time of the highest and lowest PCVs recorded are summarized in Table 6.

**Table 6:** The presence or absence of gallop sounds at the time of a cat’s highest and lowest PCV measurement for those cats where observations were made on at least 2 occasions.

		Occasion of highest PCV		
		**Gallop sound**	**No gallop sound**	**Total**
Occasion of lowest PCV	Gallop sound	7	11	18
	**No gallop sound**	2	25	27
	**Total**	9	36	45

### Complications and outcome

The median length of time that cats were in the study was 3 days (range 1-11). Intolerance to auscultation occurred in 8% (5/64; 95% CI, 2.6-17.3) cats during the study period. Forty-eight of the 64 cats (75% (95% CI, 62.6-85.0)) survived to discharge. No clinical variables at enrollment were associated with survival to discharge (Table 7). Of the non-surviving cats, 2 out of 16 died spontaneously, (12.5% (95% CI, 1.6-38.4)) the remainder were euthanized. All cats died or were euthanized due to failure to respond to treatment or perceived poor prognosis.

**Table 7:** Variables and their association with survival to discharge.

Variable	Survivors (*n* = 48)	Non-survivors (*n* = 16)	*P*-value
**Age (months)**	56 (7-211)	86 (13-192)	.336
**Bodyweight (kg)**	4.19 ± 1.06	4.05 ± 1.15	.673
**Admission PCV (%)**	18 ± 7	17 ± 6	.814
**LA:Ao**	1.49 (1.13-2.31)	1.36 (1.06-1.75)	.060
**Heart rate (bpm)**	189 ± 28	193 ± 20	.596
**Presence of a heart murmur**	60% (29/48) (95% CI, 45-74)	50% (8/16) (95% CI, 25-75)	.563
**Presence of a gallop sound**	25% (12/48) (95% CI, 14-40)	19% (3/16) (95% CI, 4-46)	.742
**Cardiac disease**	35.4% (17/48) (95% CI, 22-51)	31.2% (5/16) (95% CI, 11-59)	1.0

## Discussion

The results of this study show that heart murmurs and gallop sounds are common in anemic cats with and without cardiac disease. The likelihood of a heart murmur or gallop sound being present was significantly associated with the severity of a cat’s anemia. Having a gallop sound was also significantly associated with the presence of cardiac disease, however, having a murmur was not found to be associated with the presence of cardiac disease in this group. Caution should be applied interpreting this finding as the confidence intervals for the odds ratio were wide. This could represent a type II statistical error due to limited power to demonstrate a significant difference. It remains possible that in a larger cohort a significant association might, or might not, be found.

One of our study aims was to prospectively determine the prevalence of heart murmurs and gallop sounds in cats with anemia. Previously this has been retrospectively assessed in anemic cats presenting to an emergency room which were compared with non-anemic controls.^3^ There was a significantly higher prevalence of heart murmurs in anemic cats (30%) compared with controls (11%) and gallop sounds in anemic cats (14%) compared with controls (4%). Due to the retrospective nature of that study, a cardiac cause for the murmur was only definitively excluded in a minority of cats, with fewer than 10% undergoing echocardiography.^3^ Many apparently healthy cats (30%-51%) can have a heart murmur which could be physiological, or secondary to an undiagnosed cardiomyopathy or cardiac functional changes, such as dynamic right ventricular outflow tract obstruction.^5–9^^,^^16^

We aimed to determine the prevalence of murmurs and gallop sounds secondary to anemia by assessing the presence of cardiac disease in every cat enrolled in the study. In order to ensure the “no cardiac disease” group definitely did not have a cardiac cause for their murmur we placed cats considered to have equivocal HCM into the cardiac disease group. Consistent with the American College of Veterinary Internal Medicine (ACVIM) consensus guidelines, findings in cats with left ventricular (LV) wall thicknesses between 5 and 6 mm were interpreted in the context of body size and qualitative assessment of left atrial (LA) and LV morphology and function. The diagnosis of equivocal HCM was made by the attending board-certified cardiologist or cardiology resident in training was to ensure the “no cardiac disease” group definitely did not have a cardiac cause for their murmur. A large proportion of the cats in the “cardiac disease” group had equivocal HCM. Ideally repeat echocardiography would have been performed at a later time point to assess if these cats did truly have HCM once they had recovered from their disease process. Removing these cats from the calculation of the proportion of anemic cats with a murmur or gallop would have artificially removed equivocal cases from the classification and also would have resulted in case selection which does not accurately reflect the true spectrum of cases presented in clinical practice.

Cats can have innocent heart murmurs secondary to stress or murmurs can be inducible with pressure applied during auscultation.^5^ Both of these situations cause an increase in the turbulence of blood flow, leading to a murmur.^17^ Some cats in our study might have had murmurs for these reasons which could have changed our prevalence of murmurs secondary to anemia. It was not possible to assess for these causes. However, excluding cats that were intolerant of echocardiography and only having one investigator performing auscultation by a standard technique might have minimized the occurrence of these causes of murmurs.

Anemia causes an alteration in rheology of the blood due to a reduction in blood density and viscosity. This leads to an increase in Reynold’s number and can result in turbulent blood flow in the heart and, therefore, a heart murmur. Oxygen delivery is impaired in anemia due to the low hemoglobin concentration. To compensate for the reduced oxygen delivery, cardiac output is increased through an elevation in heart rate and stroke volume. Catecholamine release and tissue hypoxia result in tachycardia and increased inotropy. Afterload is reduced in normovolemic or hypervolemic anemia due to lower blood viscosity and vasodilation secondary to tissue hypoxia and increased nitric oxide activity. A decrease in systemic vascular resistance will lead to compensatory activation of the renin-angiotensin-aldosterone system. This leads to water and sodium retention and an increase in preload. Left ventricular filling is enhanced, leading to a larger stroke volume and cardiac output. Over time, the increase in left ventricular end-diastolic volume leads to left ventricular eccentric hypertrophy and volume overload changes.^17^^,^^18^ Chamber dilation might cause valvular insufficiency, another potential cause of a heart murmur. However, most of the cats with volume overload changes in this study did not have obvious valvular insufficiency. Therefore, altered blood rheology, and resultant increased turbulent blood flow, could be considered the most likely cause of the heart murmurs auscultated.

In our study, anemic cats with echocardiographic findings consistent with volume overload were classed as having no cardiac disease because left-sided cardiac chamber enlargement occur as a result of anemia in cats^14^^,^^18^ and dogs.^14^ Some of these changes are reversible after blood transfusion.^14^ In the cats in our cohort, the presence of a heart murmur was not consistently associated with volume overload changes, implying that the absence of a murmur cannot be interpreted to signify the absence of volume overload. This finding could support adopting a cautious approach to fluid therapy in all anemic cats to reduce the risk of iatrogenic fluid overload, whether or not a murmur is present.

A gallop sound is an additional auscultated heart sound. A wide range of estimates of the prevalence of gallop sounds are reported in cats with cardiomyopathies (2.6%-40%)^7^^,^^13^ and anemia (5.3%-60%).^1–3^ Gallop sounds can occur in people due to rapid deceleration of blood filling the ventricles^19^; this might be the cause of a gallop in anemic cats. Gallop sounds are typically thought to occur during early diastole (S3) or late diastole (S4).^19^

In our study, a significantly higher percentage of cats with cardiac disease had a gallop sound (41% (95% CI, 21-64)) compared with cats with no cardiac disease (14% (95% CI, 5-29)). A higher PCV was associated with lower odds of having a gallop sound. Overall, these results suggest that a gallop sound might be more likely to be found in an anemic cat that has concurrent cardiac disease and this could warrant judicious fluid therapy administration and consideration of further investigations for an echocardiographic abnormality.

The presence of a heart murmur, gallop sound, cardiac disease, or any other variable at presentation, were not found to be associated with survival in this study. These findings could help guide discussions with owners regarding prognosis. Individual animals should not be considered to have a lower likelihood of survival to discharge on the basis of any of these findings. Our study showed a high proportion of anemic cats had either cardiac disease (34% (95% CI, 23-47)) or volume overload changes (23% (95% CI, 14-36)). This supports exercising caution when considering the rate of administration of fluid therapy and blood transfusions to anemic cats to reduce the risk of fluid overload or transfusion associated circulatory overload.^20^

One of our hypotheses was that a change in PCV would be associated with change in murmur grade or gallop presence. This study found that the murmur grade tended to be lower when cats had a higher PCV. Also, that individual cats were more likely to have a gallop sound at their lowest PCV by comparison to their highest PCV. These findings might suggest that an increase in PCV in an individual animal could lead to an improvement in or resolution of auscultatory abnormalities. However, in our group of cats there were a large proportion that did not have serial data reported and there was a small median change in PCV over time. Findings might or might not have been different if a larger change in PCV or a greater number of cats were included in the study.

Limitations of the study include that the available period of follow up was not standardized for all cats in the study meaning that serial data are not available for all cats. This reflects the reality of recording data from cats hospitalized for variable periods and also variable tolerance of the procedures it was necessary for them to undergo. The cats enrolled in the study were presenting through our referral hospital and all required hospitalization for treatment of their condition. It is possible that animals with more severe disease might have been less tolerant of undergoing the procedures necessitated by the regular follow up required for ongoing participation in the study, leading to greater loss to follow up of cats with more severe disease.

Another limitation was that the primary investigator was not blinded to the clinical picture or PCV of the cat that was being auscultated. This could have introduced some bias into the auscultatory findings recorded. Ideally a separate blinded investigator would have performed the auscultation. Unfortunately, this was not feasible within the financial and practical constraints of the study.

Evaluating differences between groups of cats according to the presence or absence of a gallop, the presence or absence of a murmur and whether or not a cat survived to discharged involved multiple comparisons between groups for which no post hoc adjustment of the *P*-value was undertaken. Multiple comparisons risk a type I statistical error and therefore the *P*-values given in Tables 1, 3, and  5 should be interpreted with caution. The main conclusions of the study are not based on these analyses, but rather on the logistic regression analyses and the paired analyses of observations at the time of the lowest and highest PCV, so the risk of a type I statistical error with respect to the main conclusions of the study is not increased by these multiple comparisons.

Binary logistic regression models with fewer than 10 instances of the outcome of interest per predictor variable might be less reliable in their findings. For this reason, our analyses only included 2 predictor variables examining the influences of anemia severity and the presence of echocardiographically diagnosed heart disease, consistent with our original hypotheses. It is possible that other measured variables might also be associated with the likelihood of a heart murmur or gallop sound being found but our study was inadequately powered to demonstrate this. Even with the limited number of predictor variables, there were only 7.5 cats with a gallop sound per predictor variable in the model with the presence of a gallop sound as the outcome of interest meaning that the findings of this analysis should be interpreted with some caution. Some authors have suggested that the “rule of ten” can be relaxed and that in a model with between 5 and 9 events per variable only a “minor degree” of additional caution is warranted in the interpretation of the findings.^21^ Reassuringly the odds ratios for the 2 predictor variables in the multivariable analysis modeling the presence of a gallop as the outcome were similar to those obtained from univariable analyses when there were over ten events per variable (Table S1). It is also evident that the discriminatory ability of the ROC analysis for the detection of a gallop, as judged by the area under the curve, appears improved when the predicted probabilities from the binary logistic regression analysis are used rather than the PCV alone (although the 95% confidence intervals for the areas under the curve overlap).

## Conclusion

Heart murmurs and gallop sounds are common in anemic cats with and without cardiac disease. A lower PCV was associated with the presence of a heart murmur and the presence of a gallop sound. Cats with cardiac disease were not found to be more likely to have a heart murmur, but they were more likely to have an audible gallop sound.

## Supplementary Material

Supplementary_information_anemic_cats_heart_murmur_gallop_sounds_aalag261

## Data Availability

Data is available by email request to the corresponding author.

## Off-label antimicrobial declaration

Authors declare no off-label use of antimicrobials.

## Institutional animal care and use committee or other approval declaration

Approved by the Royal Veterinary College university teaching hospital Ethics and Welfare Committee (reference number URN M2023 0183). Written informed consent was obtained from the owners of every cat before inclusion in the study.

## Appendix: Materials and methods

### Cases and data collection

Client-owned cats were prospectively recruited in a referral hospital in the United Kingdom over a 1-year period (February 2024-February 2025). Written informed consent was gained from all owners for enrollment of their cat in the study. Anemic cats (PCV < 30%) without known pre-existing cardiac disease or hyperthyroidism were eligible for enrollment. Cats were excluded if owners withdrew consent, echocardiography was unable to be interpreted in light of systemic disease, if they were not tolerant of cardiac auscultation and/or echocardiography, or if they were discharged from the hospital or died before echocardiography being performed (Figure 1).

### Study protocol

After enrollment, cardiac auscultation was performed by the primary investigator (AR) once daily during hospitalization. Auscultation was performed at the following sites: left parasternal, right parasternal, and sternal points. The murmur grade or presence of a gallop sound was reported at the point of maximal intensity. The primary investigator was not blinded to the clinical picture or the PCV of the cat.

Echocardiography was performed once during hospitalization for each cat by a board-certified cardiologist or cardiology resident-in-training. Cats were gently restrained in a standing or lateral recumbency position for the scan. Echocardiography recordings were assessed by 2 cardiologists who were not blinded to the clinical picture of the cat.

Two-dimensional, M-mode and Doppler echocardiography was performed conscious using a 12.0 MHz phased array probe (GE Vivid E9 and GE Vivid iq, GE Healthcare, GE ultrasound systems, Boston, MA, USA). All left ventricular (LV) wall thickness (LVWT) and LV internal diameter (LVID) 2D measurements were measured from the right parasternal long-axis inflow (4ch RPLAX) and outflow views, and right parasternal short-axis (RPSAX) view at the level of the papillary muscles. Maximal LVWT and LVID was taken from any of these views measured at end-diastole, using a leading edge-to-leading edge technique.

Hypertrophic cardiomyopathy phenotype and equivocal HCM phenotype were defined as a LVWT of ≥6.0 mm and 5.1-5.9 mm, respectively. Left ventricular internal diameter of ≥17.0 mm was considered abnormally dilated.^14^ LA size was measured from a RPSAX view at the level of the aorta (Ao), to calculate the LA:Ao ratio measured at end-systole. An LA:Ao ratio of > 1.5 was considered abnormally dilated.^5^ M-mode of the RPSAX at the level of the papillary muscles was used to measure LV fractional shortening (LVFS). Cats with echocardiographic changes consistent with the presence of a cardiomyopathy were classified and staged as described by Luis Fuentes et al.^7^ Any cat with these findings was placed in the cardiac disease group.

Volume overload (high cardiac output) changes were defined as LA and LV dilation with normal LVWT and normal LV systolic function (LVFS > 30%). Volume overload changes were considered to be secondary to the anemia, as this has previously been described in cats, and cats with these findings were included in the “no cardiac disease” group.^13^

Cats were monitored for cardiovascular instability or stress during cardiac auscultation and echocardiography using clinical judgment and a stress score scale (Supplementary material). If a cat was deemed clinically unstable or it scored ≥3 on the scale the procedure was aborted, and the cat was excluded from ongoing procedures in the study.

### Data recording

For each day of hospitalization on which auscultation was performed, the following information was recorded: PCV within the previous 24 h, presence/absence of a murmur and, if present, murmur grade (0-VI) and presence/absence of a gallop sound. For the purposes of statistical analyses, murmur grade was treated as an ordinal variable and cats without a murmur were allocated a value of zero (0).

For each case in which there were 2 or more values of PCV recorded over the hospitalization period, the difference between the highest and lowest recorded PCV was calculated and termed “δ PCV.” Murmur grade and the presence or absence of a gallop sound at the time of the highest and lowest PCV were compared for each cat.

The cause of anemia was classified as either blood loss, hemolysis, ineffective production, or chronic disease. Outcome was reported as either survival to discharge or death during hospitalization. Deaths due to either cardiopulmonary arrest or euthanasia were grouped together as non-survival.

### Statistical analysis

Data analysis was performed, and figures were produced using statistical software (Jamovi Version 2.6.26, GraphPad Prism Version 10). Distribution of data was assessed with visual inspection of histograms and with the Shapiro–Wilk test. Summary statistics for continuous variables were reported as mean (±SD) if normally distributed or median (range) if not. Continuous and ordinal data were compared using the independent samples *t-*test or Mann–Whitney U test. Associations between categorical variables were assessed using either χ-squared or Fisher’s exact test.

Binary logistic regression analyses were undertaken, one with the presence of a gallop sound as the outcome variable and the other with the presence of a heart murmur as the outcome variable. In both, admission PCV and the presence or absence of echocardiographically diagnosed heart disease were used as the predictor variables. ROC analysis was performed to evaluate the diagnostic accuracy of admission PCV and predicted probabilities from the logistic regression models in the detection of animals with a murmur or gallop respectively. The AUC was calculated and, where appropriate, the optimal cut-off point was determined by Youden’s index.

In cats in which physical examination findings and PCV were noted on at least 2 occasions, observations recorded at the time of the lowest PCV were compared to those observed at the time of the highest PCV. Murmur grade was compared using Wilcoxon Signed Rank test and the presence of a gallop sound was compared using McNemar test. Significance level was set at a *P*-value of <.05 for all tests.

Ethical approval was gained from the Royal Veterinary College university teaching hospital Ethics and Welfare Committee (URN: M2023 0183). A grant was provided by the Royal Veterinary College Animal Care Trust (VPO 6313) to fund echocardiography scans and provide these at no charge to the clients owning the cats.

## Abbreviations

bpm: beats per minute
HCM: hypertrophic cardiomyopathy
LVWT: left ventricular wall thickness
LA:Ao: left atrial to aorta ratio
PCV: packed cell volume
ROC: receiver operator curve
