# Supplementary material for: Prospective evaluation of the prevalence and characteristics of heart murmurs and gallop sounds in anemic cats
Source: J Vet Intern Med. 2026 Oct 24;40(5):aalag261. doi: 10.1093/jvimsj/aalag261 (PMC13695151; doi:10.1093/jvimsj/aalag261)
Supplement: Supplementary file 1 [file supplementary_information_anemic_cats_heart_murmur_gallop_sounds_aalag261.docx]

**Supplementary information anemic cats heart murmur gallop sounds**

**SUPPLEMENTARY INFORMATION**

**Materials and methods**

**Stress score scale**

<https://familypethealth.com/understanding-fas-fear-anxiety-and-stress-in-the-vet-clinic/#:~:text=All%20of%20these%20signs%20of,of%20your%20pet%27s%20emotional%20health>)

**Results**

|  |  |  | **95% Confidence Interval (CI)** | |
| --- | --- | --- | --- | --- |
| Predictor | p value | Odds ratio (OR) | Lower | Upper |
| PCV at admission | 0.010 | 0.867 | 0.778 | 0.966 |
| Cardiac disease: Yes/No | 0.021 | 4.154 | 1.2360 | 13.960 |

**Supplementary Table 1.** Results demonstrating the association of PCV at admission and the presence or absence of cardiac disease as predictors taken from univariable binary logistic regression analyses with the presence of a gallop sound (yes/no) as the outcome variable.
